# Computational, crystallographic studies, cytotoxicity and anti-tubercular activity of substituted 7-methoxy-indolizine analogues

**DOI:** 10.1371/journal.pone.0217270

**Published:** 2019-06-04

**Authors:** Katharigatta Narayanaswamy Venugopala, Sandeep Chandrashekharappa, Melendhran Pillay, Hassan H. Abdallah, Fawzi M. Mahomoodally, Subhrajyoti Bhandary, Deepak Chopra, Mahesh Attimarad, Bandar E. Aldhubiab, Anroop B. Nair, Nagaraja Sreeharsha, Mohamed A. Morsy, Shinu Pottathil, Rashmi Venugopala, Bharti Odhav, Koleka Mlisana

**Affiliations:** 1 Department of Pharmaceutical Sciences, College of Clinical Pharmacy, King Faisal University, Al-Ahsa, Kingdom of Saudi Arabia; 2 Department of Biotechnology and Food Technology, Durban University of Technology, Durban, South Africa; 3 Institute for Stem Cell Biology and Regenerative Medicine, NCBS, TIFR, GKVK, Bangalore, India; 4 Department of Microbiology, National Health Laboratory Services, KZN Academic Complex, Inkosi Albert Luthuli Central Hospital, Durban, South Africa; 5 School of Pharmacy, Universiti Sains Malaysia, Penang, Malaysia; 6 Chemistry Department, College of Education, Salahaddin University, Erbil, Iraq; 7 Department of Health Sciences, Faculty of Science, University of Mauritius, Réduit, Mauritius; 8 Department of Chemistry, Indian Institute of Science Education and Research Bhopal, Bhauri, Bhopal, Madhya Pradesh, India; 9 Department of Pharmacology, Faculty of Medicine, Minia University, El-Minia, Egypt; 10 Department of Biomedical Sciences, College of Clinical Pharmacy, King Faisal University, Al-Ahsa, Kingdom of Saudi Arabia; 11 Department of Public Health Medicine, University of KwaZulu-Natal, Howard College Campus, Durban, South Africa; Aligarh Muslim University, INDIA

## Abstract

Indolizines are heteroaromatic compounds, and their synthetic analogues have reportedly showed promising pharmacological properties. In this study, a series of synthetic 7-methoxy-indolizine derivatives were synthesised, characterised and evaluated for *in vitro* whole-cell anti-tuberculosis (TB) screening against susceptible (H37Rv) and multi-drug-resistant (MDR) strains of *Mycobacterium tuberculosis* (MTB) using the resazurin microplate assay method. The cytotoxicity was evaluated using the MTT assay. *In silico* molecular-docking study was conducted for compounds **5a-j** against enoyl-[acyl-carrier] protein reductase, a key enzyme of the type II fatty acid synthesis that has attracted much interest for the development of novel anti-TB compounds. Thereafter, molecular dynamic (MD) simulation was undertaken for the most active inhibitors. Compounds **5i** and **5j** with the methoxy functional group at the meta position of the benzoyl group, which was at the third position of the indolizine nucleus, demonstrated encouraging anti-TB activity against MDR strains of MTB at 16 μg/mL. *In silico* studies showed binding affinity within the range of 7.07–8.57 kcal/mol, with **5i** showing the highest binding affinity. Hydrogen bonding, π-π- interactions, and electrostatic interactions were common with the active site. Most of these interactions occurred with the catalytic amino acids (Pro193, Tyr158, Phe149, and Lys165). MD simulation showed that **5j** possessed the highest binding affinity toward the enzyme, according to the two calculation methods (MM/PBSA and MM/GBSA). The single-crystal X-ray studies of compounds **5c** and **5d** revealed that the molecular arrangements in these two structures were mostly guided by C-H···O hydrogen-bonded dimeric motifs and C-H···N hydrogen bonds, while various secondary interactions (such as π···π and C-H···F) also contributed to crystal formation. Compounds **5a**, **5c**, **5i**, and **5j** exhibited no toxicity up to 500 μg/mL. In conclusion, **5i** and **5j** are promising anti-TB compounds that have shown high affinity based on docking and MD simulation results.

## Introduction

**Fig 1:**
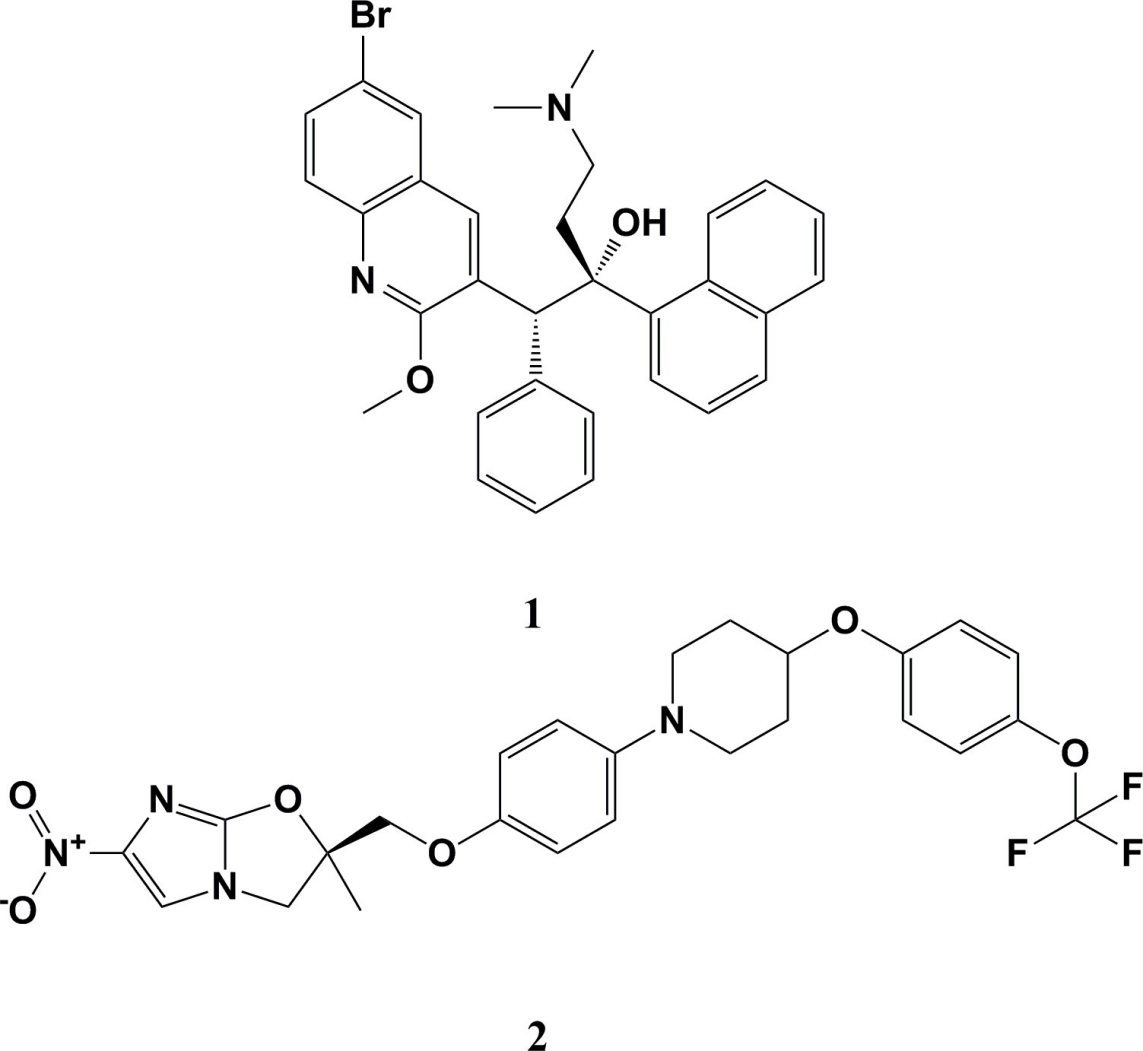
Chemical structure of clinically approved anti-TB drugs bedaquiline (1) and delamanid (2).

*Mycobacterium tuberculosis* (MTB) is the bacterial pathogen that underlies the infectious disease known as tuberculosis (TB). This disease affects the lungs and a number of other body systems and structures. According to WHO 2018 report, TB resulted in nearly 1.3 million deaths in those who are HIV-negative, and in 300,000 deaths among those who are HIV-positive [1]. Every year, new TB cases are reported worldwide and human immunodeficiency virus (HIV)-infected persons are up to 37 times more vulnerable to developing TB [2]. The development of multi-drug-resistant (MDR)-TB, extensively drug-resistant (XDR)-TB, and totally drug-resistant (TDR)-TB [3], as well as co-infections with acquired immunodeficiency syndrome (AIDS) and the risks involved in cases of TB among patients with diabetes mellitus [4], has resulted in a grave situation worldwide. Treating MDR-TB and XDR-TB is difficult, as second-line drugs have become far less effective [5]. This problem has been made worse by the evolution of TDR MTB strains [6] that are untreatable using the existing arsenal of anti-TB drugs. Based on the last 40 years of academic and pharmaceutical industry inventions, only bedaquiline (**1**) was the first novel anti-TB drug permitted by the United States Food and Drug Administration (US FDA) authority in December 2012 for the treatment of MDR-TB [7], while delamanid (**2**) was the second anti-TB agent to be approved by the European Medicines Agency (EMA) in late 2013 [8] (Fig 1).

**Fig 2:**
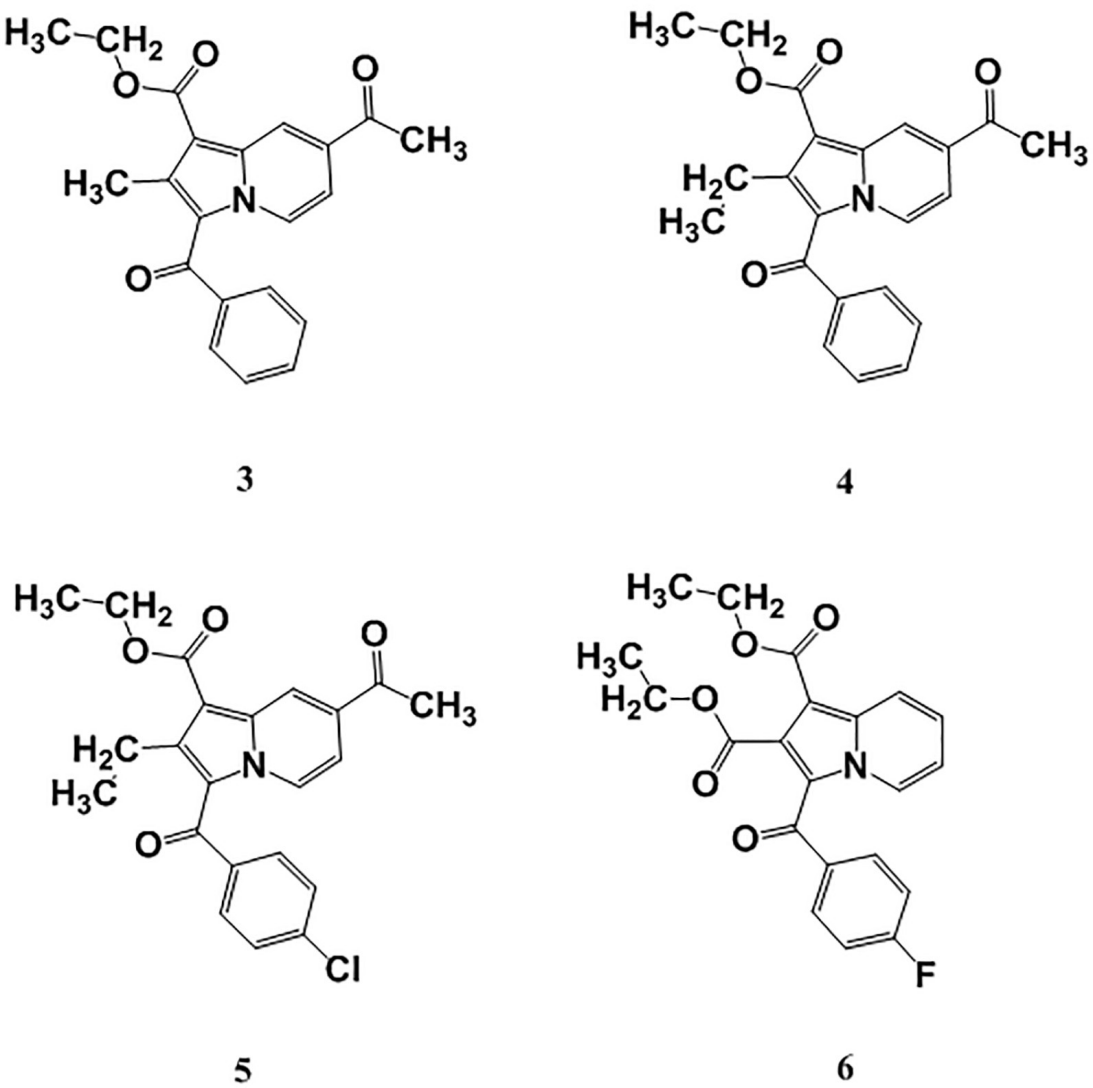
Molecular structure of compounds ethyl 7-acetyl-3-benzoyl-2-methyl-indolizine-1-carboxylate (3), ethyl 7-acetyl-3-benzoyl-2-ethyl-indolizine-1-carboxylate (4), ethyl 7-acetyl-3-(4-chlorobenzoyl)-2-ethyl-indolizine-1-carboxylate (5), and diethyl 3-(4-fluorobenzoyl)indolizine-1,2-dicarboxylate (6) for their anti-TB activity against MDR strains of MTB [10].

**Fig 3:**
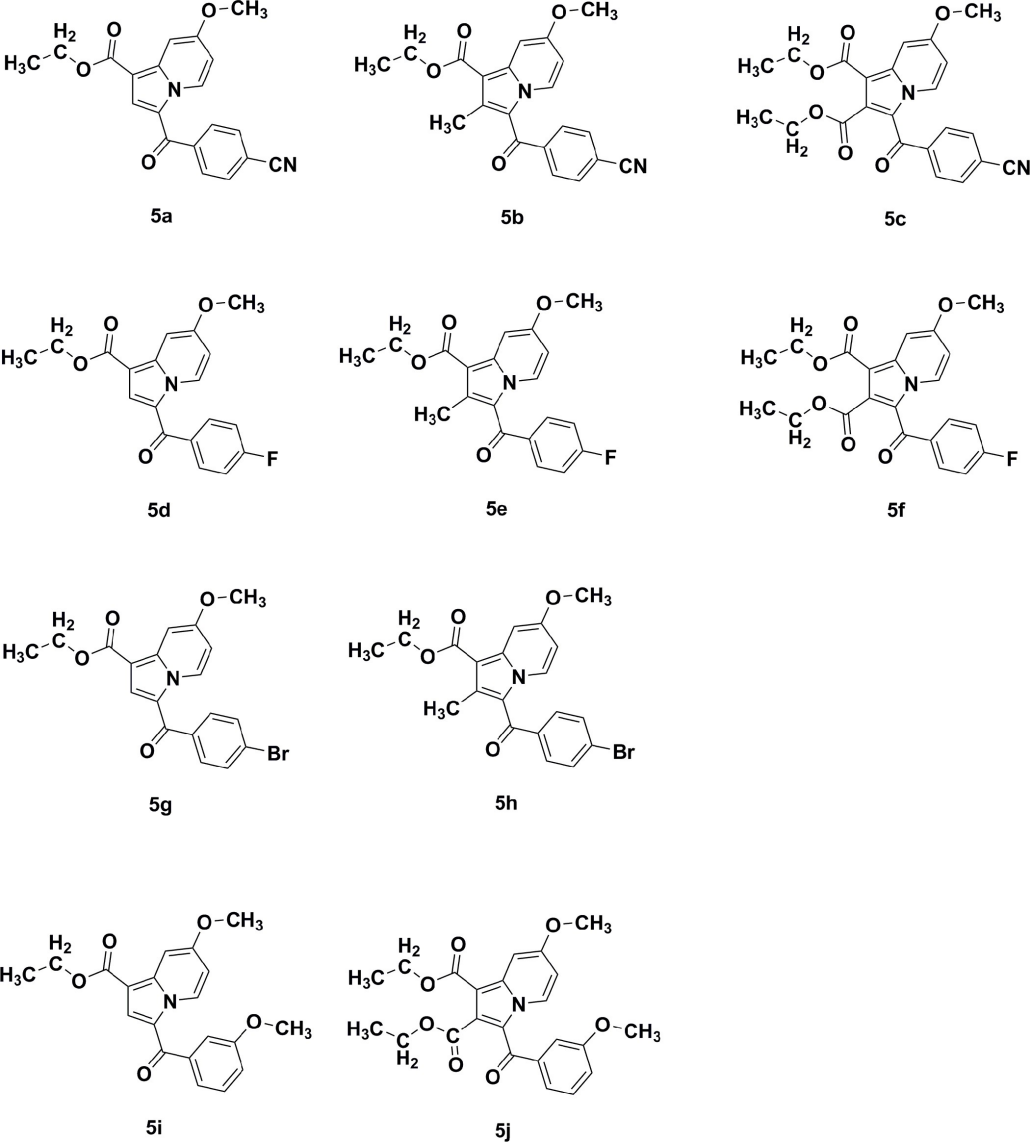
Chemical structures of substituted 7-methoxy-indolizine analogues tested for their anti-TB activity against H37Rv and MDR MTB strains.

Fatty-acid biosynthesis is critical in the synthesis of the mycobacterial cell wall. The enoyl-[acyl-carrier] protein reductase enzyme elongates fatty-acid chains. Further, it acts as a catalyst to reduce α- and β-unsaturated fatty acids that are in complex with the enzyme, and has become of great interest when developing synthetic indolizine compounds that demonstrate anti-TB activity [9, 10]. Indolizines are heteroaromatic compounds, and their synthetic analogues have reportedly demonstrated promising pharmacological properties [11]. Specifically, they have exhibited analgesic [12], anticancer [13, 14], antidiabetic [15], antihistaminic [16], anti-inflammatory [17, 18], antileishmanial [19], antimicrobial [20], antimutagenic [21], antioxidant [22], antitubercular [10, 23], antiviral [24], larvicidal [25], and herbicidal activities [26]. In continuation of our previous work aimed at developing such synthetic indolizine analogues as enoyl-[acyl-carrier] protein reductase enzyme inhibitors (Fig 2), we undertake the screening of substituted 7-methoxy-indolizine analogues (Fig 3) to determine their whole-cell anti-TB properties against H3Rv and MDR strains of MTB using the resazurin microplate assay (REMA) plate method.

**Fig 4:**
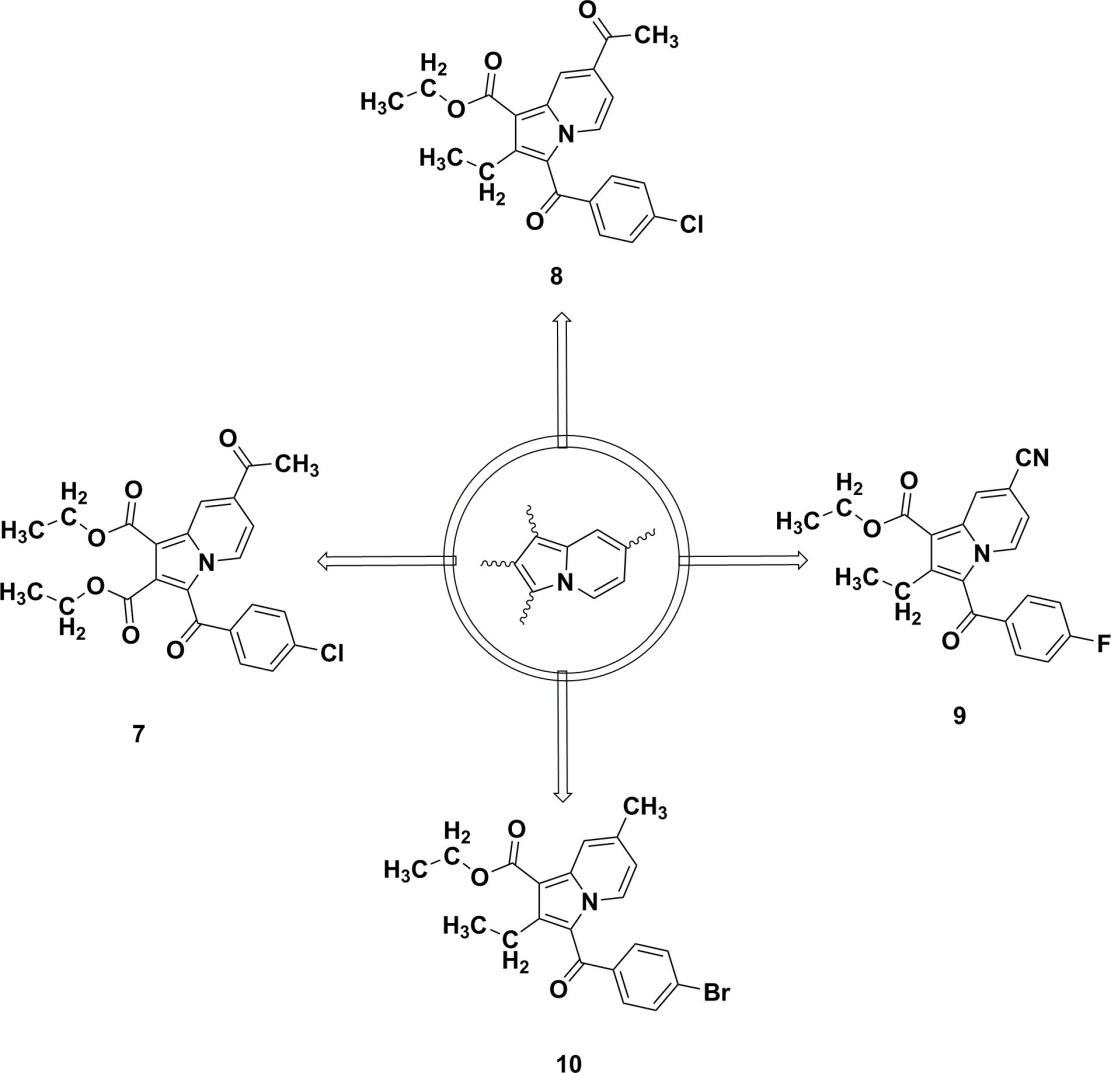
Indolizine lead compounds identified for their anticancer (7) [14] and anti-tubercular (8) properties [10] against MDR strains of MTB, as well as for their COX-2 inhibition (9) [18] and larvicidal activity (10) [25] against *Anopheles arabiensis*.

Our group recently investigated various substituted indolizine scaffolds for their synthesis, crystallography, and pharmacological properties, including their anticancer properties [14], their larvicidal activity against *Anopheles arabiensis* [25, 27], and their cyclooxygenase-2 (COX-2) inhibition properties (Fig 4) [18, 28].

Owing to an urgent call for the development of novel scaffold as anti-TB agents, we recently launched a medicinal chemistry program aimed at developing novel, natural, cyclic depsi-peptides [29] and heterocyclic scaffolds as potential anti-TB agents [10, 30–32]. We previously reported the anti-TB activity of indolizines [10], where a series of tri-substituted indolizines were identified as promising anti-TB agents. Among them, indolizine **8** (Fig 4) was found to be potent against H37Rv and MDR strains of MTB. Based on this observation we envisaged to synthesize and test 7-methoxy-indolizine analogues (**5a-j**) for anti-tubercular properties against H37Rv and MDR MTB strains.

## Materials and methods

### Materials

All chemicals reported here were obtained from Sigma-Aldrich Co. (St. Louis, MO, USA), while the solvents were obtained from MilliporeSigma (Burlington, MA, USA). Thin-layer chromatography (TLC) was employed to observe chemical reactions, and this process was performed on silica gel (Sigma-Aldrich Co.) on aluminum foil; n-hexane and ethyl acetate (4:6) were used as the solvent. The reactions were visualized under an ultraviolet (UV)-light/iodine chamber. A Büchi melting point B-545 apparatus was used to measure the melting points (Büchi, Labortechnik, Flawil, Switzerland). Infrared (IR) spectra were recorded on a Nicolet 6700 Fourier-transform infrared (FT-IR) spectrometer. Further, ^1^H and ^13^C-NMR spectra were recorded using Bruker AVANCE III 400 MHz (Bruker Corporation, Billerica, MA, USA) with CDCl_3_ (solvent). Chemical shifts (*δ*) were indicated in ppm, with tetramethylsilane (TMS) as a reference; coupling constants (*J*) were recorded (Hz). The splitting pattern was documented as follows: *s*, singlet; *d*, doublet; *q*, quartet; and *m*, multiplet. Liquid chromatography (LC)-mass spectrometry (MS) (Agilent 1100 series) was used to measure the mass spectra, in conjunction with MSD and 0.1% aqueous trifluoroacetic acid in an acetonitrile system on the C18-BDS column. Elemental analysis was conducted using a FLASH EA 1112 CHN analyzer (Thermo Finnigan LLC, New York, NY, USA).

#### Synthesis of 1-(2-(4-substituted phenyl)-2-oxoethyl)-4-methoxypyridin-1-ium bromide (3a–3c)

**Fig 5:**
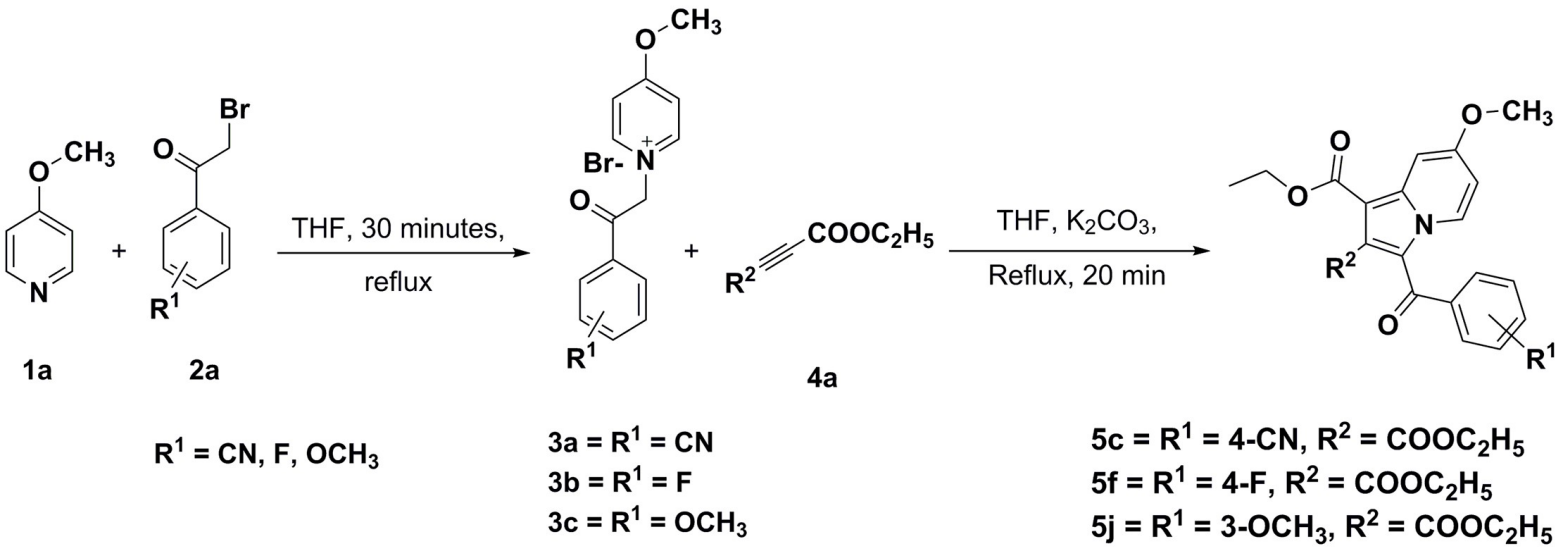
Synthetic scheme for the construction of substituted 7-methoxy-indolizine analogues.

4-Substituedphenacylbromide (**1a**) (0.0091 mol) was added to a well-stirred solution of 4-methoxypyridine (**2a**) (0.0091 mol) in dry tetrahydrofuran (12 mL) at ambient temperature; and the resulting reaction mixture was refluxed for 30 minutes (Fig 5) and monitored on TLC. The separated product was filtered, recrystallized using ethanol as a solvent, and dried at room temperature to afford a 98%–99% yield of 1-(2-(4-substitutedphenyl)-2-oxoethyl)-4-methoxypyridin-1-ium bromide (**3a–3c**).

#### 1-(2-(4-cyanophenyl)-2-oxoethyl)-4-methoxypyridin-1-ium bromide (3a)

Appearance: Yellow-colored product. Yield, 98%. ^1^H-NMR (400MHz, DMSO-*d*_6_) *δ* = 8.96–8.94 (d, *J* = 7.2 Hz, 2H), 8.52–8.50 (d, *J* = 7.0 Hz, 2H), 7.90–7.88 (d, *J* = 7.2 Hz, 2H), 7.58–7.56 (d, *J* = 8 Hz, 2H), 6.29 (s, 2H), 4.14 (s, 3H); LC-MS (ESI, Positive): *m/z*: (M+H)^+^: 253.2.

#### 1-(2-(4-fluorophenyl)-2-oxoethyl)-4-methoxypyridin-1-ium bromide (3b)

Appearance: White-colored product. Yield, 99%. ^1^H-NMR (400MHz, DMSO-*d*_6_) *δ* = 8.72–8.70 (d, *J* = 7.0 Hz, 2H), 8.75–8.67 (d, *J* = 7.2 Hz, 2H), 8.30–8.27 (m, 2H), 8.13–8.10 (t, *J* = 8.8 Hz, 2H), 6.28 (s, 2H), 4.19 (s, 3H); LC-MS (ESI, Positive): *m/z*: (M+H)^+^: 246.12.

#### 4-Methoxy-1-(2-(3-methoxyphenyl)-2-oxoethyl) pyridin-1-ium bromide (3c)

Appearance: Yellow-colored product. Yield, 99%. ^1^H-NMR (400MHz, DMSO-*d*_6_) *δ* = 9.02–9.00 (d, *J* = 7.2 Hz, 2H), 8.54–8.52 (d, *J* = 7.2 Hz, 2H), 7.49–7.47 (d, *J* = 7.2 Hz, 1H), 7.36–7.31 (t, *J* = 7.2 Hz, 1H), 7.15 (s, 1H), 7.10–7.08 (d, *J* = 7.2 Hz, 1H), 6.27 (s, 2H), 4.10 (s, 3H), 3.88 (s, 3H); LC-MS (ESI, Positive): *m/z*: (M+H)^+^: 258.2.

### Synthetic procedure for the preparation of diethyl 3-(4-cyanobenzoyl)-7-methoxyindolizine-1,2-dicarboxylate (5c) [28]

A mixture of diethyl but-2-ynedioate (**4a**) (0.1 mmol) and K_2_CO_3_ (0.1 mmol) was added to a stirred solution of 1-(2-(4-cyanophenyl)-2-oxoethyl)-4-methoxypyridin-1-ium bromide (**3a**) (0.1 mmol) in dry tetrahydrofuran (15 mL). The reaction medium was refluxed for 20 minutes and reaction completion was monitored on TLC. Once the reaction was complete, the solvent was removed and diluted with ethyl acetate. The organic layer was rinsed with water and brine and dried over sodium sulfate. The obtained residue was purified by column chromatography to afford a 79% yield of diethyl 3-(4-cyanobenzoyl)-7-methoxyindolizine-1,2-dicarboxylate (**5c**). Title compounds **5f** and **5j** were synthesized following the same procedure; Table 1 outlines the physicochemical constants of the characterized title compounds.

**Table 1:** Physicochemical parameters of ethyl 7-methoxy-3-(substituted benzoyl)-indolizine-1-carboxylate 5a–5j.

Compound code	Mol formulae (Mol Mass)	R^1^	R^2^	Yield (%)^a^	m.p (°C) reported	m.p (°C) found	cLog*P*^b^
**5a**	C_20_H_16_N_2_O_4_ (348)	4-CN	H	81	165	165	3.9570
**5b**	C_21_H_18_N_2_O_4_ (362)	4-CN	CH_3_	74	191	192	4.4560
**5c**	C_23_H_20_N_2_O_6_ (420)	4-CN	COOC_2_H_5_	79	-	171	3.4454
**5d**	C_19_H_16_FNO_4_ (341)	4-F	H	80	118	118	4.5293
**5e**	C_20_H_18_FNO_4_ (355)	4-F	CH_3_	73	137	138	5.0283
**5f**	C_22_H_20_FNO_6_ (413)	4-F	COOC_2_H_5_	79	-	147	4.0199
**5g**	C_19_H_16_BrNO_4_ (402)	4-Br	H	75	183	182	5.2493
**5h**	C_20_H_18_BrNO_4_ (416)	4-Br	CH_3_	77	148	148	5.7483
**5i**	C_20_H_19_NO_5_ (353)	3-OCH_3_	H	85	116	116	4.4986
**5j**	C_23_H_23_NO_7_ (425)	3-OCH_3_	COOC_2_H_5_	76	-	142	4.0294

^*a*^ Yields calculated after purification by column chromatography.

^*b*^ ChemDraw Professional 16 was used to calculate cLog*P* of the title compounds.

Title compounds **5a**, **5b**, **5d**, **5e**, **5g**, **5h**, and **5i** were prepared and the physicochemical constants were compared with a previous report [18] and tabulated in Table 1. The molecular structures of the test compounds are illustrated in Fig 3.

#### Diethyl 3-(4-cyanobenzoyl)-7-methoxyindolizine-1,2-dicarboxylate (5c)

Appearance: Brown crystalline compound; Fourier transform (FT)-IR (KBr) cm^-1^ = 2987, 2229, 1737, 1693, 1647, 1596. ^1^H-NMR (400 MHz CDCl_3_) *δ =* 9.55–9.53 (d, *J* = 7.2Hz, 1H), 7.78 (s, 1H), 7.61–7.55 (m, 4H), 6.82–6.79 (m, 1H), 4.36–4.31 (q, *J* = 7.2Hz, 2H), 3.99 (s, 3H), 3.76–3.70 (q, *J* = 7.2Hz, 2H), 1.36–1.33 (t, *J* = 7.2Hz, 3H), 1.14–1.10 (t, *J* = 7.2Hz, 3H). ^13^C-NMR (100 MHz CDCl_3_) *δ =* 184.89, 164.98, 163.21, 160.09, 141.57, 138.57, 132.53, 131.21, 130.23, 130.05, 126.28, 119.40, 110.24, 102.64, 97.66, 61.77, 60.28, 55.83, 14.23, 13.60. LC-MS (ESI, Positive): *m/z* = (M+H)^+^: 421.2; Anal. calculated for C_23_H_20_N_2_O_6_; C, 65.71; H, 4.79; N, 6.66; Found; C, 65.70; H, 4.81; N, 6.62.

#### Diethyl 3-(4-fluorobenzoyl)-7-methoxyindolizine-1,2-dicarboxylate (5f)

Appearance: Yellow amorphous compound; FT-IR (KBr) cm^-1^ = 2981, 1737, 1699, 1647, 1607, 1230. ^1^H-NMR (400 MHz CDCl_3_) *δ* = 9.50–9.48 (d, *J* = 7.2Hz, 1H), 7.77–7.71 (m, 3H), 7.15–7.11 (m, 2H), 6.81–6.78 (m, 1H), 4.36–4.31 (q, *J* = 7.2Hz, 2H), 3.98 (s, 3H), 3.75–3.69 (q, *J* = 7.2Hz, 2H), 1.36–1.33 (t, *J* = 7.2Hz, 3H), 1.13–1.10 (t, *J* = 7.2Hz, 3H). ^13^C-NMR (100 MHz CDCl_3_) *δ =* 184.77, 166.11, 165.02, 163.60, 163.27, 159.96, 141.47, 136.04, 136.00, 132.25, 131.23, 131.14, 129.96, 119.58, 115.59, 114.97, 110.15, 102.44, 97.59, 61.68, 60.25, 55.81, 14.23, 13.62. LC-MS (ESI Positive): *m/z =* (M+H)^+^: 414.12; Anal. calculated for C_22_H_20_FNO_6_: C, 63.92; H, 4.88; N, 3,39; Found; C, 63.98; H, 4.85; N, 3.40.

#### Diethyl 7-methoxy-3-(3-methoxybenzoyl)indolizine-1,2-dicarboxylate (5j)

Appearance: Light-yellow crystalline compound; FT-IR (KBr) cm^-1^ = 2981, 1738, 1693, 1647, 1608. ^1^H-NMR (400 MHz CDCl_3_) *δ* = 9.56–9.54 (d, *J* = 7.2Hz, 1H), 7.78 (s, 1H), 7.38–7.36 (m, 1H), 7.29–7.28 (m, 1H), 7.21 (s, 1H), 7.10–7.08 (m, 1H), 6.80–6.78 (m, 1H), 4.35–4.30 (q, *J* = 7.2Hz, 2H), 3.99 (s, 3H), 3.81 (s, 3H), 3.73–3.68 (q, *J* = 7.2Hz, 2H), 1.36–1.32 (t, *J* = 7.2Hz, 3H), 1.11–1.08 (t, *J* = 7.2Hz, 3H). ^13^C-NMR (100 MHz CDCl_3_) *δ* = 185.90, 165.08, 163.35, 159.91, 159.09, 141.46, 141.00, 132.45, 130.10, 129.14, 121.14, 119.73, 118.38, 112.99, 110.08, 102.45, 97.57, 61.63, 60.21, 55.80, 55.36, 14.22, 13.54. LC-MS (ESI Positive): *m/z* = (M+H)^+^: 426.14. Anal. calculated for C_23_H_23_NO_7;_ C, 64.93, H, 5.45, N, 3,29; Found; C, 64.95, H, 5.42, N, 3.32.

### Crystallography

#### Crystal growth, single-crystal data collection, and refinement details

Suitable single crystals of compounds **5c** and **5d** were grown individually from the slow evaporation of toluene at ambient conditions. Single-crystal X-ray diffraction of **5c** and **5d** was performed using a Bruker SMART APEX II diffractometer with Mo-Kα radiation (χ = 0.71073 Å). Data collection was performed when the temperature reached 110 (2) K using an Oxford Cryostream cooling system (Bruker Apex II software) [33]. The Bruker SAINT software program was used to conduct cell refinement and data reduction [34]. SADABS was used for absorption correction [35], while the structures were solved using SHELXS-97 [36] and refined using full-matrix least-squares methods based on F^2^ (SHELXL-2018) [37] (WinGX software program, version 2014.1) [38]. The hydrogen atoms were refined using a riding model (Uiso(H) = 1.2Ueq [C_aromatic_] and *U*_iso_(H) = 1.5*U*eq [methyl groups]). One of the–OC_2_H_5_ groups (attached to the C19 atom) for the **5c** structure was refined as a two-component positional disorder with the occupancy of 83:17. Geometric calculations were carried out using PLATON [39]. ORTEP and packing diagrams were created using the Mercury 3.5.1 (CCDC) program [40]. The crystal data and the structure refinement parameters are given in Table 2.

**Table 2:** Single crystal data and structure refinement parameters for 5c and 5d.

Identification code	5c	5d
CCDC number	1873349	1873348
Empirical formula	C_23_ H_20_ N_2_ O_6_	C_19_ H_16_ F N O_4_
Formula weight	420.41	341.33
Temperature	110(2) K	110(2) K
Wavelength	0.71073 Å	0.71073 Å
Crystal system	Monoclinic	Triclinic
Space group	*P*2_1_/*n*	*P*-1
Unit cell dimensions	a = 12.1530(12) Å.	a = 4.1368(3) Å
	b = 17.6845(15) Å.	b = 11.9682(8) Å
	c = 19.1511(19) Å.	c = 16.5416(10) Å
	α = 90°	α = 74.912(4)°
	β = 99.051(4)°	β = 88.675(5)°
	γ = 90°	γ = 80.110(5)°
Volume	4064.7(7) Å3	778.79(9) Å3
Z	8	2
Density (calculated)	1.374 Mg/m3	1.456 Mg/m3
Absorption coefficient	0.101 mm-1	0.110 mm-1
F(000)	1760	356
Crystal size	0.280 x 0.160 x 0.060 mm3	0.320 x 0.120 x 0.060 mm3
Theta range for data collection	1.861 to 27.875°.	5.017 to 29.575°.
Index ranges	-15< = h< = 13, -23< = k< = 23, -25< = l< = 21	-5< = h< = 5, -15< = k< = 16, -22< = l< = 21
Reflections collected	30715	10474
Independent reflections	9438 [R(int) = 0.0794]	4218 [R(int) = 0.0601]
Completeness to theta = 25.242°	98.6%	97.3%
Absorption correction	Semi-empirical from equivalents	Semi-empirical from equivalents
Max. and min. transmission	0.7460 and 0.6507	0.7460 and 0.6647
Refinement method	Full-matrix least-squares on F2	Full-matrix least-squares on F2
Data / restraints / parameters	9438 / 3 / 577	4218 / 0 / 228
Goodness-of-fit on F2	1.010	1.051
Final R indices [I>2sigma(I)]	R1 = 0.0715, wR2 = 0.1684	R1 = 0.0580, wR2 = 0.1404
R indices (all data)	R1 = 0.1296, wR2 = 0.1958	R1 = 0.1010, wR2 = 0.1627
Largest diff. peak and hole	0.318 and -0.266 e.Å-3	0.267 and -0.293 e.Å-3

### Computational studies

#### Molecular-docking study

The three-dimensional (3D) molecular structures of the studied compounds (**5a–5j**) were built using Gaussview and optimized using an AM1 semi-empirical method to their ground state level using Gaussian09 [41]. The crystal structure of the enoyl-[acyl-carrier] protein reductase enzyme was downloaded from the Protein Databank RCSB (PDB; PDB code entry: 1ZID), in which the enzyme was crystalized with an isonicotinic acyl NADH inhibitor. To prepare the protein for docking, water molecules, inhibitor molecules, and any co-crystalized molecules were removed. Autodock 4 [42] was used to dock the studied compounds at the active site of the enzyme. First, the crystalized inhibitor was docked to verify the docking procedure and to confirm the position of the active site; the remaining compounds used the same procedure, in which Kollman-united atom charges neutralized the enzyme with a grid box of 60×60×60 with 0.375 Å distance between points. Then, 250 runs for each inhibitor were carried out using a Lamarckian genetic algorithm. The docked conformations were clustered and ranked according the binding free energy. Discovery Studio 5.0 visualizer was used to visualize the best docked poses and to elucidate the intra-molecular interactions at the enzyme’s active site.

#### Molecular-dynamics simulation

Amber14 was used to perform all simulations for the enzyme-inhibitor complex immersed in a water box using a TIP3P explicit solvent; an ff14SB force field was employed at a temperature equal to 300 K [43]. Four sodium ions were added to neutralize this system. The system was minimized in two steps, followed by 2.0 fs time step simulations with a cutoff of 10 Å for non-bonded interactions. Short simulation with a constant-volume periodic boundary was performed to increase the temperature from 0 K to 300 K, followed by 3.5 ns of a constant-pressure periodic boundary MD at 300 K using the Langevin thermostat. The same procedure was performed for the enzyme complex and the most active ligands (**5i** and **5j**), as well as for the enzyme that did not have any inhibitor (for comparative purposes).

### Antitubercular activity

#### Resazurin microplate assay (REMA)

Anti-TB screening of test compounds **5a–j** was performed using the colorimetric REMA plate method [31, 44].

#### Determining the minimum inhibitory concentration (MIC)

All test compounds (**5a–j**) were further evaluated by the agar incorporation method, which was performed three times, and which targeted an H37Rv strain and an MDR-TB strain (isoniazid = 0.2 μg/mL and rifampicin >1.0 μg/mL). MIC determination was performed [45], with some modifications. A Level II Biosafety laboratory was used to carry out this experiment. MTB reference strain H37Rv (American Type Culture Collection [ATCC], Manassas, VA, USA: 25177) and MDR-TB were cultured in Middlebrook 7H11 medium for a total of 3 weeks [46]. The strain was supplemented with OADC (0.005%, v/v, oleic acid; 0.2%, w/v, glucose; 0.085%, w/v, NaCl; 0.02%, v/v, catalase; and 0.5%, 171 w/v, bovine serum albumin [BSA]), and incubated at a temperature of 37°C. Fresh cultures were used to in the preparation of a standardized inoculum in a sterile tube containing 0.05% Tween 80 and 4.5 mL of phosphate buffer; (5 mm in diameter) were used for vortexing. The bacterial supernatant was then standardized to McFarland Number 1 with water, yielding a bacterial concentration of ~1×10^7^cfu/mL. The bacterial suspension was diluted with water; then, a total of 100 μL of the dilution was placed onto Middlebrook 7H10 agar plates containing drug doses ranging from 8–0.125 μg/mL (to begin, 8 μg/mL of the drug was dissolved in distilled water and then diluted twofold to reach the desired concentration before being added to the agar medium). The MICs of the drugs (i.e., that inhibited >1% of the organism’s growth when compared with controls) were obtained 3 weeks following incubation. Table 3 presents the anti-TB results when compared with H37Rv (ATCC: 25177), MDR-MTB, and XDR-MTB.

**Table 3:** In vitro whole-cell anti-TB activity of 7-methoxy-indolizine analogues (5a–j) against H37RV and MDR-MTB isolates.

Compound Code	Anti-TB activity—MIC (μg/mL)	
	H37RV isolate	MDR-MTB isolate*
**5a**	8	32
**5b**	NA	NA
**5c**	32	64
**5d**	8	NA
**5e**	32	NA
**5f**	32	NA
**5g**	NA	NA
**5h**	NA	NA
**5i**	8	16
**5j**	8	16

MIC, minimum inhibitory concentration.

*These isolates were found to be resistant to the first-line antibiotics rifampicin (1 μg/mL), and isoniazid (0.2 μg/mL).

NA: not active

### Safety studies- cytotoxicity assay

Title compounds **5a**, **5c**, **5i**, and **5j** which exhibited anti-TB activity against MDR strains of MTB, were subjected to safety studies by 3-(4,5-dimethylthiazol-2-yl) -2,5-diphenyltetrazolium bromide (MTT) assay. The MTT cytotoxicity assay is used to evaluate the cytotoxic effects of the most promising compounds against peripheral blood mononuclear cells (PBMCs)–i.e., **5a**, **5c**, **5i**, and **5j** –according to the described protocol [47].

## Results and discussion

### Chemistry

To explore the role of various functional groups on the indolizine nucleus, a series of indolizine scaffolds were synthesized using a greener synthetic approach and the yield was found to be in the range of 73%–85% following purification by column chromatography. The synthetic scheme for the construction of the title compounds (**5a–j**) is illustrated in Fig 5, and the physicochemical characteristic details are tabulated in Table 1. The intermediates required to develop novel title compounds **5c**, **5f**, and **5j** were synthesized between 92%–99% yield and the characterization details are listed under the experimental section. Title compounds **5a–c, 5d–f**, and **5g–h** were prepared with nitrile, fluoro, and bromo functional groups, respectively, at the para position of the benzoyl group at the third position of the indolizine nucleus. Compounds **5i** and **5j** were prepared by having a methoxy functional group at the meta position of the benzoyl group, which is at the third position of the indolizine nucleus. Compounds **5a**, **5d**, **5g**, and **5i** were unsubstituted at the second position of the indolizine nucleus, whereas compounds **5b**, **5e**, and **5h** had a methyl substituent at the second position of the indolizine nucleus. Conversely, compounds **5c**, **5f**, and **5j** had an ethyl ester functional group at the second position of the indolizine nucleus. The molecular structure of the resynthesized compounds **5a**, **5b**, **5d**, **5e**, **5g**, **5h**, and **5i** was confirmed by LC-MS and melting-point determination. Novel compounds **5c**, **5f**, and **5j** were prepared using a green chemistry approach, and their molecular structures were confirmed by FT-IR, NMR (^1^H and ^13^C), LC-MS, and elemental analysis. FT-IR spectra of the title compounds **5c**, **5f**, and **5j** exhibited carbonyl stretching at the 1737–1738 cm^**–**1^ range. In the case of proton nuclear magnetic resonance (NMR) spectra, the singlet peak for the methoxy group was observed at 3.98–3.99 ppm. During ^13^C NMR, carbonyl carbon stretching for compounds **5c**, **5f**, and **5j** was observed in the range of *δ* = 184.77–184.89. The molecular ion peaks of the compounds observed on LC-MS were in alignment with their molecular mass, whereby the results were within ±0.4% of the calculated theoretical values. *c*Log*P* of the title compounds was calculated using ChemDraw Prof version 16.0 and the calculated results were 3.9570–5.7483. The bromo group at the para position of the benzoyl group, which is at the third position of the indolizine nucleus, exhibited the highest *c*Log*P* value at 5.7483. Selected title compounds **5c** and **5d** were subjected to single-crystal X-ray studies and crystal data were deposited into the Cambridge Crystallographic Data Centre (CCDC; numbers 1873349 and 1873348, respectively).

### Crystallography

#### Analysis of the crystal structures of compounds 5c and 5d

**Fig 6:**
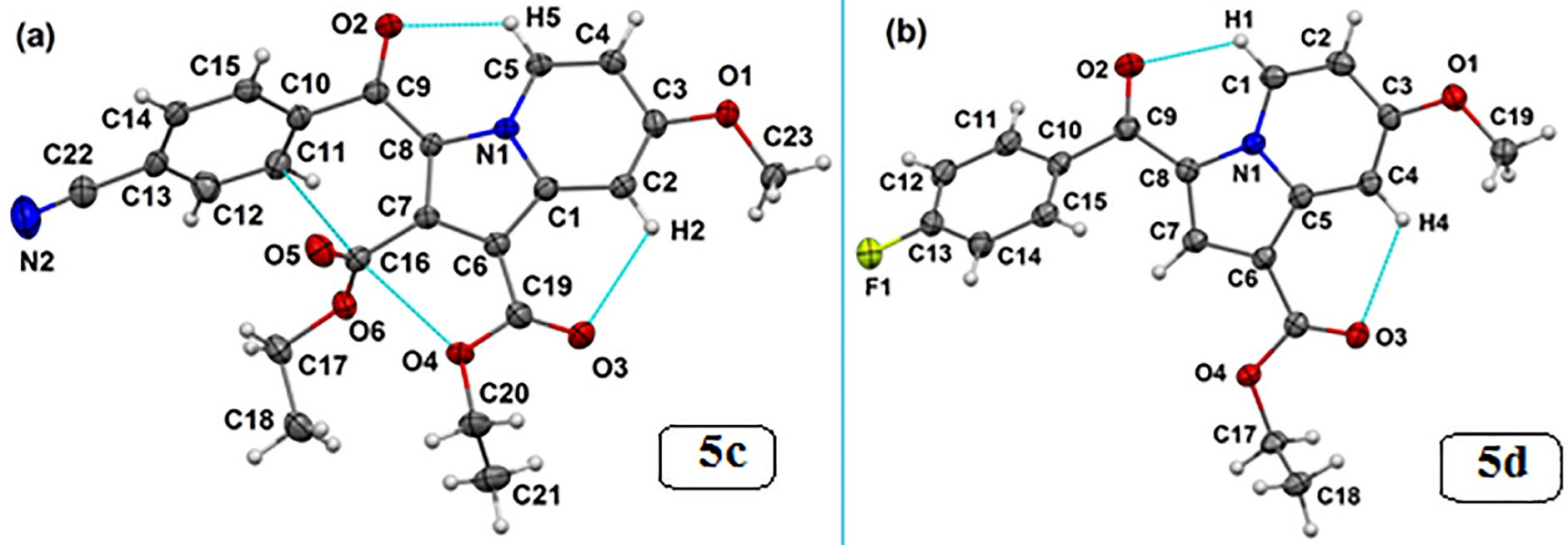
Thermal ellipsoidal plots drawn at the 50% probability level for the crystal structures of (a) 5c (second symmetry independent molecule has been omitted for clarity, Z' = 2) and (b) 5d. Dotted lines indicate intramolecular interactions.

**Fig 7:**
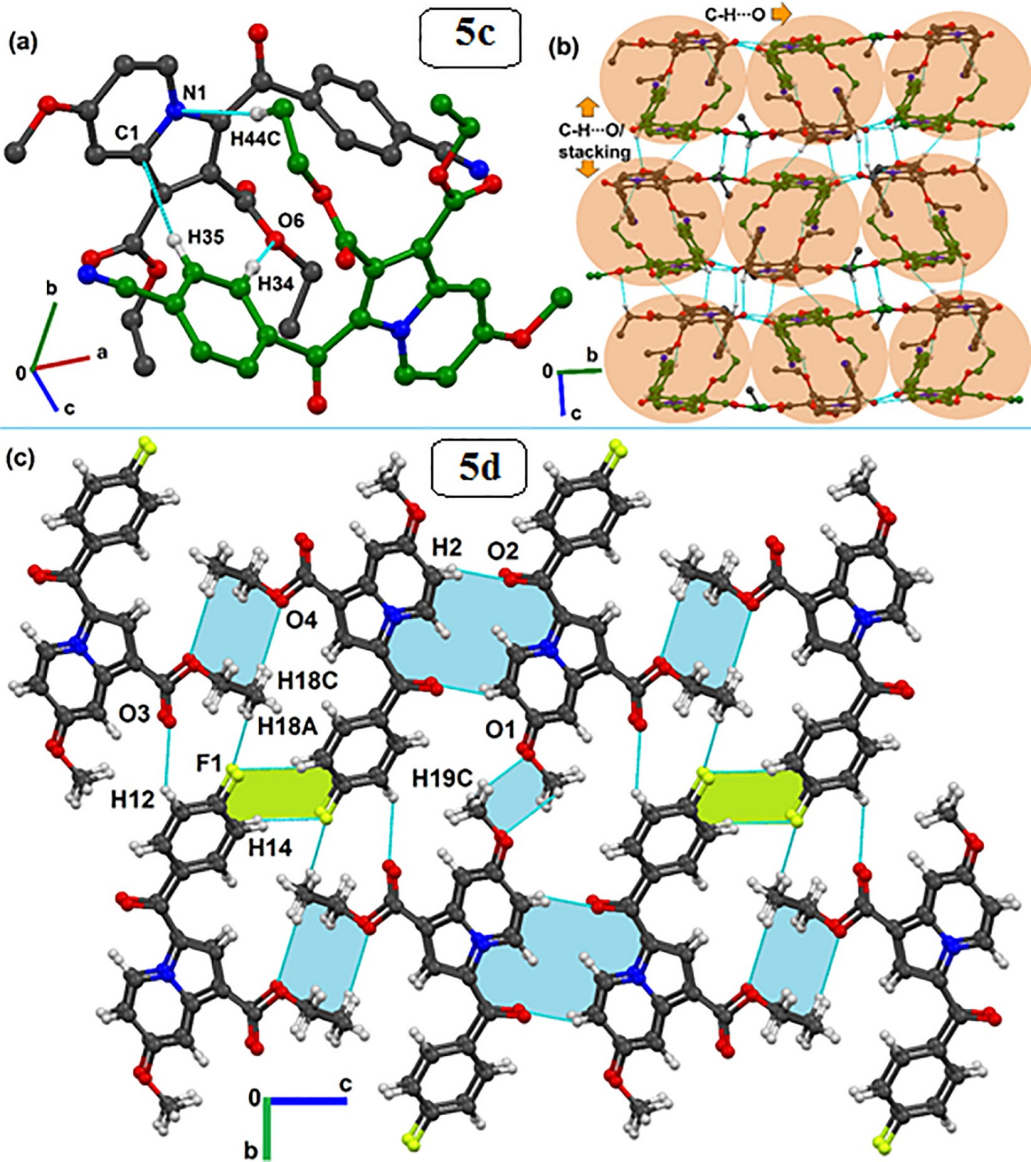
Formation of (a) dimer by two symmetry-independent molecules (black and green) in the asymmetric unit of 5c utilizing C-H···O, C-H···N and C-H···π interactions. (b) Crystal packing for 5c molecules via the association of dimeric motifs (light red) through C-H···O and π···π stacking interactions. (c) Packing arrangement of 5d molecules stabilized via various strong to weak C-H···O (light blue) and C-H···F (light green) dimers. Different-colored carbon atoms indicate different symmetry-independent molecules. Non-interacting hydrogen atoms were removed in the case of 5d to clarify the packing view.

The single-crystal X-ray diffraction study for the title compound revealed that **5c** crystallizes in the monoclinic *P*2_1_/*n* space group with two symmetrical free molecules (Z′ = 2), while the **5d** crystallizes in the *P*-1 space group of the triclinic crystal system, consisting of one molecule (Z′ = 1) in the asymmetric unit (Table 3). The molecular structures of both **5c** and **5d** are shown in Fig 6, which depicts that the molecular conformation in the crystal of **5c** is primarily stabilized via intramolecular C-H···O, C···O, and C···C(π) contacts. Similarly, intramolecular C-H···O contacts lock the crystal conformation in molecule **5d**. Looking for the supramolecular structure of **5c**, the presence of two symmetry-independent molecules forms a dimeric motif, which is stabilized by C-H···O, C-H···N and C-H···π hydrogen bonds (see molecules in black and green; Fig 7A). Such hydrogen-bonded dimers (light-red shaded circle in Fig 7B) further extend along the *b*-crystallographic direction via a C-H···O hydrogen bond, forming a layer of molecules. These molecular layers are assembled through C-H···O and π···π stacking interactions in a parallel manner (see shaded circles) along the crystallographic *c*-direction to complete the two-dimensional crystal structure of **5c**. Conversely, the crystal structure formed by **5d** molecules is primarily governed by C-H···O hydrogen-bonded dimers (light-blue shade, Fig 7C).

In addition, weak C-H···F (light-green) dimers also provide further support in the formation of molecular sheet-like supramolecular constructs down the *bc*-crystallographic plane. These molecular sheets are further stacked along the *a*-direction (Fig 7C). The geometrical parameters of all possible intra- and intermolecular interactions for both **5c** and **5d** crystal structures are provided in Table 4.

**Table 4:** List of intra- and intermolecular interactions present in compounds 5c and 5d.

Motifs	D–H…A	Symmetry	Geometry		
			D…A/Å	H…A/ Å	∠D–H…A/°
5c [C1 > C23 –first molecule; C24 > C46 –second molecule]					
**I**	C2-H2···O3	x, y, z (intra)	3.028(2)	2.45	112
	C5-H5···O2		2.862(2)	2.21	117
	C16···O4		2.724(2)	-	-
	C16···C11(π)		3.073(2)	-	-
	C24-H24···O8		2.868(2)	2.21	117
	C27-H27···O9		3.007(2)	2.41	113
	C42···O10		2.793(2)	-	-
	C42···C34(π)		3.026(2)		
**II**	C11-H11···O12	x, y, z	3.739(3)	2.66	175
	C12-H12···O9		3.528(3)	2.71	132
	C23-H23C···N4		3.761(3)	2.71	164
	C17-H17A···N3		3.565(3)	2.77	137
	C34-H34···O6		3.535(3)	2.51	157
	C44-H44C···N1		3.672(3)	2.61	168
	C35-H35···C2(π)		3.927(3)	2.87	168
	C12-H12···C27(π)		4.008(3)	2.96	164
**III**	C25-H25···O2	-x+1/2, y-1/2, -z+1/2	3.393(2)	2.31	178
	C14-H14···O8		3.212(2)	2.61	115
	C12-H12···C14(π)		3.746(2)	2.70	162
**IV**	C23-H23A···O11	x-1/2, -y+1/2, z-1/2	3.356(2)	2.32	159
	C20-H20B···O9		3.265(2)	2.53	161
	π ···π (molecular stacking)		3.994(3)	-	-
**V**	C23-H23B···O5	x-1, y, z	3.530(3)	2.51	157
	C43-H43B···N2		3.554(3)	2.76	131
**VI**	C4-H4···O8	-x-1/2, y+1/2, -z+1/2	3.801(2)	2.81	153
	C37-H37···O2		3.455(2)	2.45	154
**VII**	C45-H45B···O11	x+1, y, z	3.563(2)	2.56	154
	O7···C37(π)		3.003(2)	-	-
**VIII**	C15-H15···O2	-x, -y+1, -z	3.489(3)	2.48	155
	C15-H15···C5(π)		3.728(3)	2.88	135
**IX**	C38-H38···O1	x+1/2, -y+1/2, z+1/2	3.441(2)	2.58	136
**X**	C41-H41C···O10	-x,-y+1,-z+1	3.616(2)	2.61	155
**5d**					
**I**	C1-H1···O2	x, y, z (intra)	2.930(2)	2.30	115
	C4-H4···O3		3.066(2)	2.48	113
**II**	C18-H18···F1	x+1, y-1, z	3.618(3)	2.55	172
	C12-H12···O3		4.664(3)	2.61	145
**III**	C17-H17A···F1	x, y+1, z	3.295(3)	2.47	133
**IV**	C17-H17B···O4	-x+2, -y+1, -z+1	3.546(2)	2.88	119
	C18-H18C···O4		2.492(2)	2.84	119
**V**	C17-H17A···O3	x+1, y, z	3.552(3)	2.70	136
	C17-H17B···O4		3.548(3)	2.88	120
**VI**	C14-H14···F1	-x+2, -y, -z+1	3.333(2)	2.43	140
**VII**	C19-H19A···O3	x-1, y, z	3.782(3)	2.80	152
	C17-H17A···O3		3.552(3)	2.70	136
	π ···π (molecular stacking)		3.632- 4.137(3)	-	-
**VIII**	C19-H19B···O1	- x-1, -y+2, -z	3.364(2)	2.43	143
**IX**	C2-H2···O2	-x, -y+1, -z	3.377(2)	2.33	162

### Computational studies

#### Docking calculations

**Fig 8:**
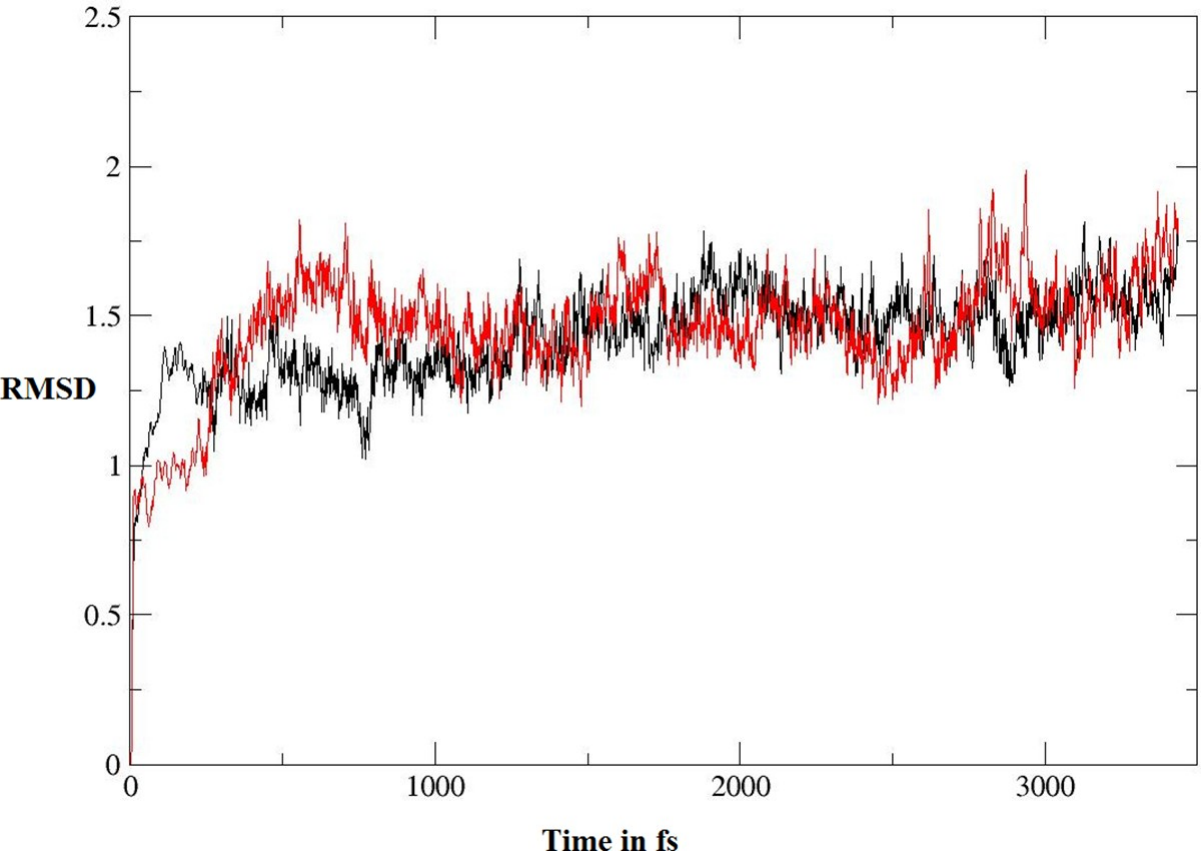
Intermolecular interactions of docked 7-methoxy-indolizine analogues 5a–5j at the active site of the enoyl-[acyl-carrier] protein-reductase enzyme.

To investigate the binding affinity of the studied compounds and to establish potential correlations between the experimental results, a docking study was performed at the active site of the enzyme. The binding affinity and predicted inhibition constant are summarized in Table 5. Indeed, the 10 derivatives showed binding affinity within the range of 7.07–8.57 kcal/mol, with **5i** showing the highest. Obviously, the non-bonding interactions between the ligands and amino acids at the active site are responsible for the formation of a stable enzyme-inhibitor complex. For that, the formed complexes with the best binding affinity were visualized and the interactions with the active site were investigated (the interactions are detailed in Fig 8). Hydrogen bonding, pi-pi interactions, and electrostatic interactions are common at the active site. In addition, most of these interactions occurred with the active site residues Pro193, Tyr158, Phe149, and Lys165.

**Table 5:** Docking free energy and estimated inhibition constant (K_i_) of the docked indolizine analogues 5a–j.

Indolizine analogues	Docking free energy	Inhibition constant
**5a**	–8.46 kcal/mol	633.99 nM
**5b**	–8.39 kcal/mol	703.54 nM
**5c**	–7.86 kcal/mol	1.72 uM
**5d**	–7.90 kcal/mol	1.62 uM
**5e**	–8.47 kcal/mol	617.21 nM
**5f**	–7.07 kcal/mol	6.57 uM
**5g**	–8.54 kcal/mol	547.97 nM
**5h**	–8.53 kcal/mol	563.15 nM
**5i**	–8.57 kcal/mol	525.81 nM
**5j**	–7.36 kcal/mol	4.01 uM

#### Molecular dynamic simulations

**Fig 9:**
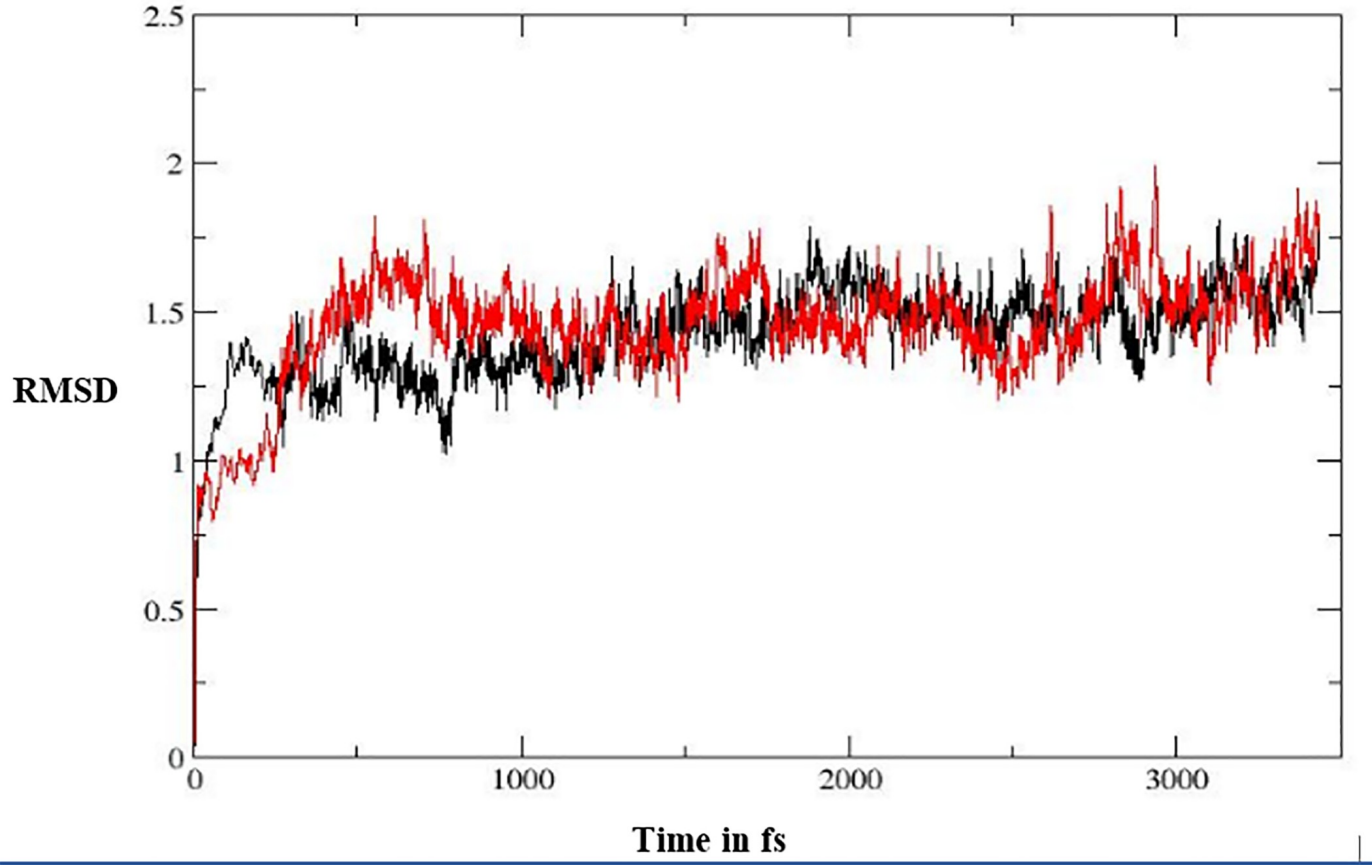
RMSD graph computed for 3.5 ns; the black line represents compound 5i and the red line represents compound 5j.

**Fig 10:**
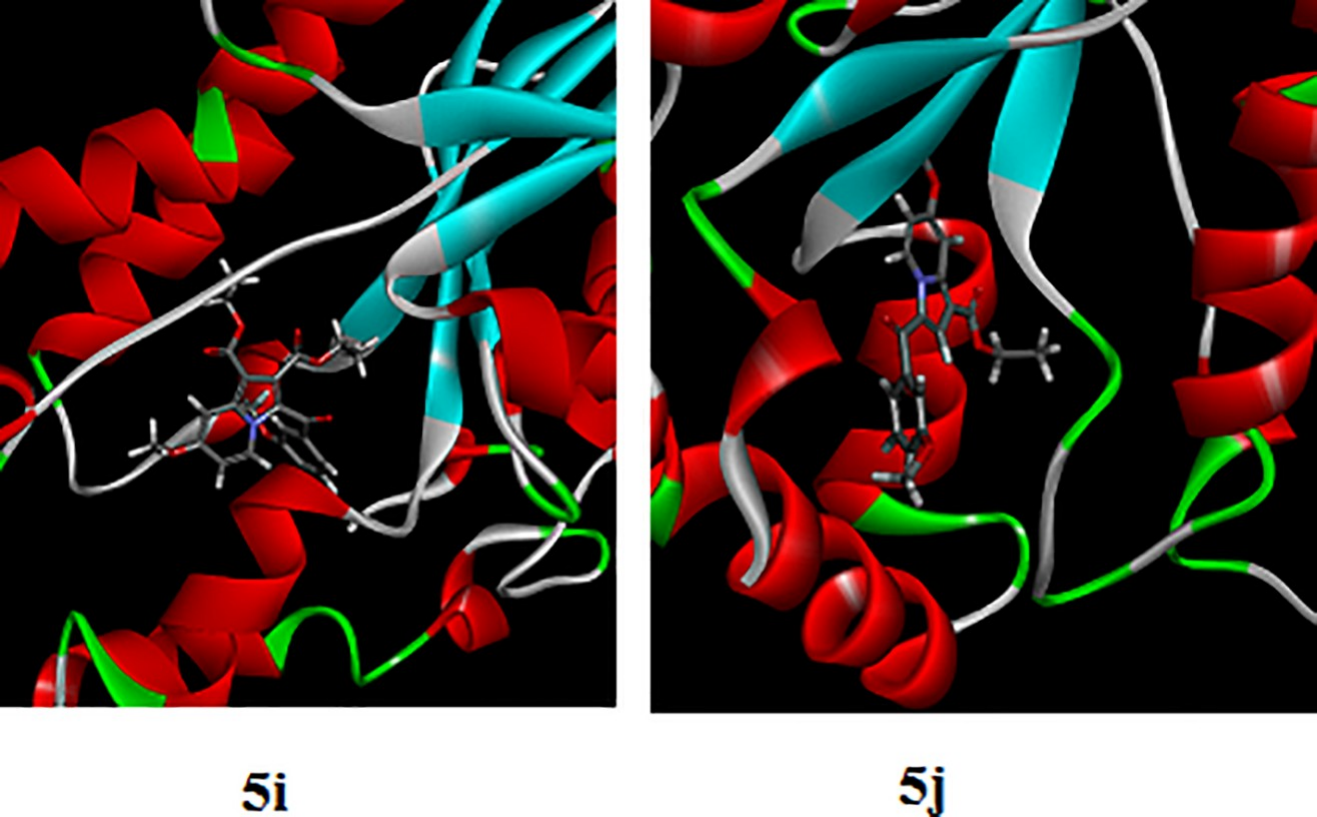
The interaction of ligands 5i and 5j at the active site of the enoyl-[acyl-carrier] protein-reductase enzyme following simulation.

As shown in Table 2, **5I** and **5J** showed the highest anti-TB biological activity, which was supported by the results of the docking study. To study the stability of the complexes of these compounds with the active site, molecular-dynamic simulation was performed for 3.5 ns (Fig 9). At the beginning, the complexes were optimized, relaxed, and equilibrated followed by long simulation for around 3.0 ns. Following the stability of the simulated complexes, molecular mechanics/Poisson–Boltzmann surface area (MM/PBSA) and molecular mechanics/generalized born surface area (MM/GBSA) were computed; these are widely used to estimate the free binding energy, as shown in Table 6. It was clear that **5j** showed higher binding affinity toward the enzyme, according to the two methods of calculation (MM/PBSA and MM/GBSA), which is in contrast with the docking results. It is well known that the docking process is performed while the structure of the enzyme is fixed. In contrast, as the MM/PBSA and MM/GBSA calculations are based on the molecular dynamic simulation, the enzyme-inhibitor complex is flexible which may enhance the reliability of results. Fig 10 illustrates the interaction of ligands **5i** and **5j** at the active site of the enoyl-[acyl-carrier] protein reductase enzyme following the simulation.

**Table 6:** MM/PBSA and MM/GBSA calculations for compounds 5i and 5j.

Compound	ΔG (MM/PBSA)	ΔG (MM/GBSA)
**5i**	–5.803	–18.841
**5j**	–7.004	–21.346

### Anti-TB activity

Title compounds **5a**–**5j** were evaluated for their MIC against H37Rv and MDR strains of MTB (Table 2). Compounds **5i** and **5j** have a methoxy group at the meta position of the benzoyl group, which was at the third position of the indolizine nucleus; they exhibited similar anti-TB activity at 8 μg/mL against the H37Rv strain and at 16 μg/mL against the MDR strains of MTB. Compound **5a**, which has a nitrile group at the fourth position of the benzoyl group, revealed activity at 8 μg/mL against H37Rv and at 32 μg/mL against MDR strains of MTB. However, di-ethyl ester functional groups on the indolizine nucleus in compound **5c** with the nitrile group at the fourth position of the benzoyl group showed anti-TB activity at 8 μg/mL against H37Rv and at 32 μg/mL against MDR strains of MTB. Although compounds **5e** and **5f** exhibited anti-TB activity against the susceptible H37Rv strain at 32 μg/mL, they failed to show anti-TB activity against MDR strains of MTB; meanwhile, compounds **5b**, **5g**, and **5h** exhibited no activity against either of the anti-TB strains in the experiment.

### Safety studies

The anti-TB test compounds **5a**, **5c**, **5i**, and **5j** from the series in Fig 5 were evaluated in safety studies by MTT assay. It was found that test compounds **5a**, **5c**, **5i**, and **5j** exhibited no toxicity up to 500 μg/mL across PBM cell lines.

## Conclusions

Indolizine compounds were previously identified as a class of anti-TB agents against MDR strains of MTB. Here, we presented our medicinal chemistry efforts that were aimed at screening indolizine analogues with various functional groups to determine their anti-TB activity in vitro. We performed computational docking for the compounds **5a** to **5j** and dynamics simulations for the compounds **5i** and **5j**, and we also detailed the crystallographic insights of two selected compounds that had different substituents to assess the role of inter- and intra-molecular interactions. Compounds **5i** and **5j** emerged as promising anti-TB agents against MDR strains of MTB with no toxicity up to 500μg/mL. Docking and MD-simulation results tended to support the corresponding observed biological activity; these data showed that **5j** has higher binding affinity when compared with **5i**. The findings of the crystallographic analysis clearly suggest that the molecular arrangements of the **5c** and **5d** structures are mostly guided by C-H···O hydrogen-bonded dimeric motifs and C-H···N hydrogen bonds, while various secondary interactions (including π···π and C-H···F) were also found to contribute to the crystal formation.

## Supporting information

S1 Fig FT-IR of diethyl 3-(4-cyanobenzoyl)-7-methoxyindolizine-1,2-dicarboxylate (5c). (TIF)

S2 Fig ^1^H-NMR of diethyl 3-(4-cyanobenzoyl)-7-methoxyindolizine-1,2-dicarboxylate (5c). (TIF)

S3 Fig ^13^C-NMR of diethyl 3-(4-cyanobenzoyl)-7-methoxyindolizine-1,2-dicarboxylate (5c). (TIF)

S4 Fig FT-IR of diethyl 3-(4-fluorobenzoyl)-7-methoxyindolizine-1,2-dicarboxylate (5f). (TIF)

S5 Fig ^1^H-NMR of diethyl 3-(4-fluorobenzoyl)-7-methoxyindolizine-1,2-dicarboxylate (5f). (TIF)

S6 Fig ^13^C-NMR of diethyl 3-(4-fluorobenzoyl)-7-methoxyindolizine-1,2-dicarboxylate (5f). (TIF)

S7 Fig FT-IR of diethyl 7-methoxy-3-(3-methoxybenzoyl)indolizine-1,2-dicarboxylate (5j). (TIF)

S8 Fig ^1^H-NMR of diethyl 7-methoxy-3-(3-methoxybenzoyl)indolizine-1,2-dicarboxylate (5j). (TIF)

S9 Fig -NMR of diethyl 7-methoxy-3-(3-methoxybenzoyl)indolizine-1,2-dicarboxylate (5j). (TIF)

S10 Fig CheckCIF of diethyl 3-(4-cyanobenzoyl)-7-methoxyindolizine-1,2-dicarboxylate (5c). (TIF)

S11 Fig CheckCIF of ethyl 3-(4-fluorobenzoyl)-7-methoxyindolizine-1-carboxylate (5d). (TIF)

## Abbreviations

MDR-TB: multi-drug-resistant tuberculosis
INH: isoniazid
TB: tuberculosis
TDR: totally drug resistant
XDR-TB: extensively drug resistant
MM/PBSA: molecular mechanics/Poisson–Boltzmann surface area
MM/GBSA: Molecular mechanics/Generalized Born surface area
MIC: minimum inhibitory concentration
REMA: resazurin microplate assay
DMF: Dimethylformamide
MTB: *Mycobacterium tuberculosis*
