# Supplementary figures and images for: Computational, crystallographic studies, cytotoxicity and anti-tubercular activity of substituted 7-methoxy-indolizine analogues
Source: PLoS One. 2019 Jun 4;14(6):e0217270. doi: 10.1371/journal.pone.0217270 (PMC6548424; doi:10.1371/journal.pone.0217270)

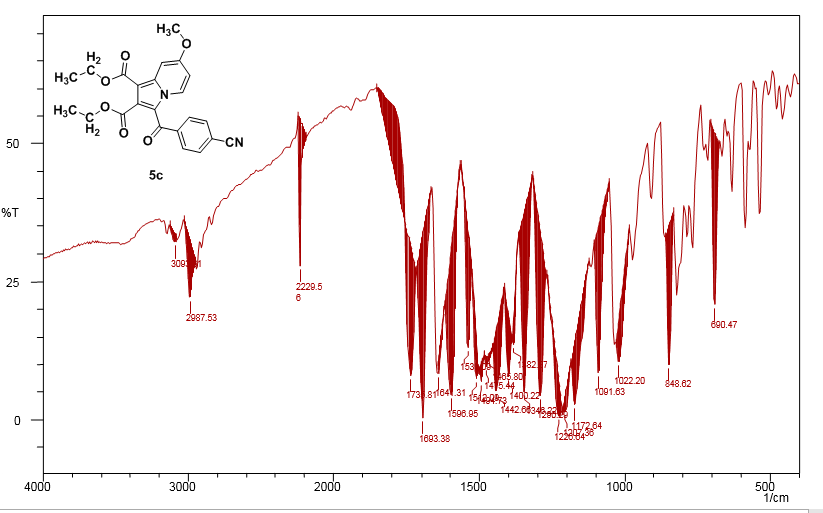

Supplement: S1 Fig — (TIF) [file pone.0217270.s001.tif]

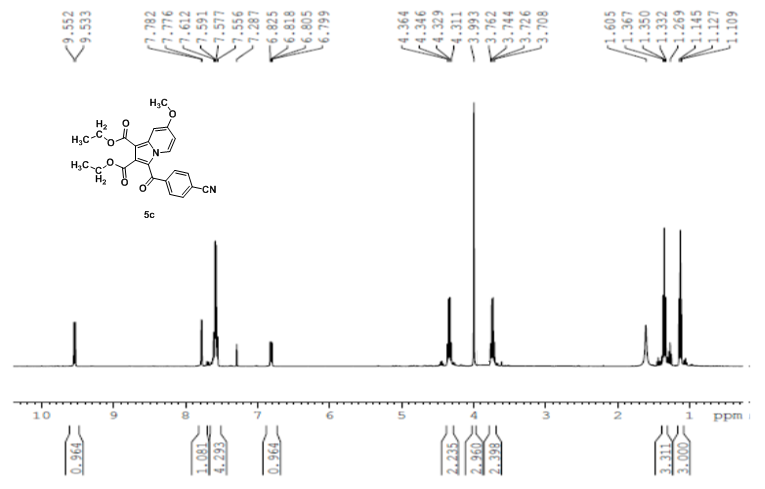

Supplement: S2 Fig — (TIF) [file pone.0217270.s002.tif]

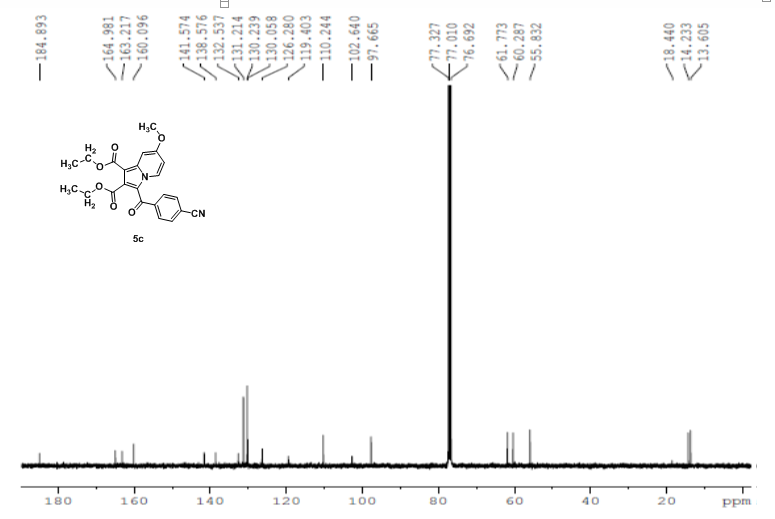

Supplement: S3 Fig — (TIF) [file pone.0217270.s003.tif]

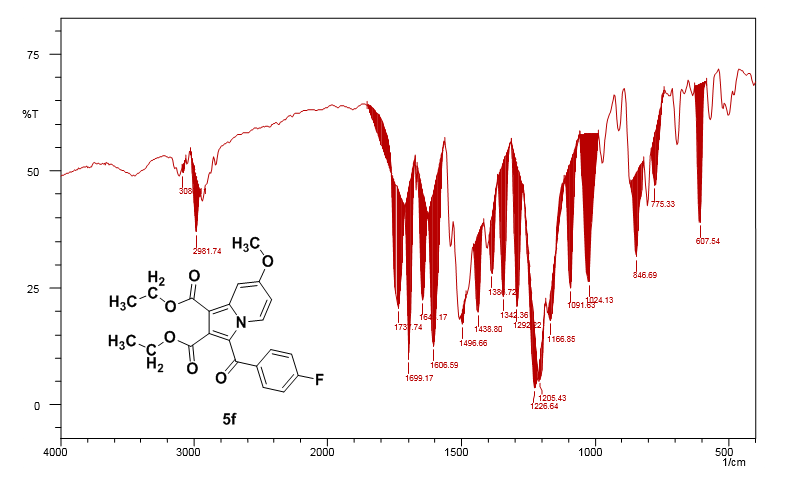

Supplement: S4 Fig — (TIF) [file pone.0217270.s004.tif]

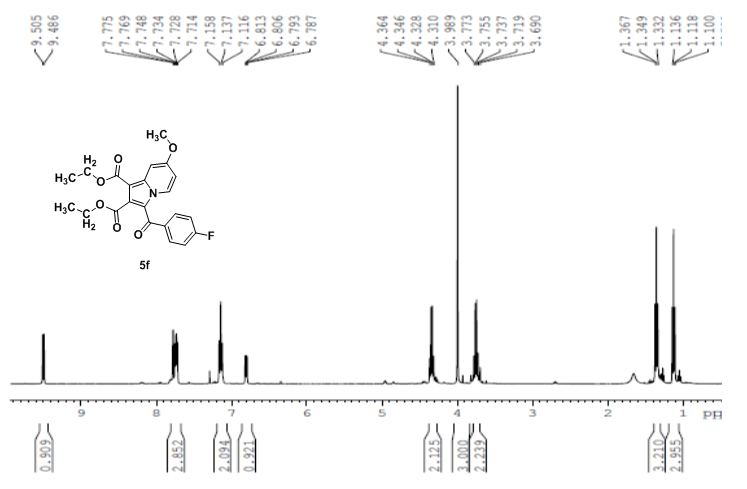

Supplement: S5 Fig — (TIF) [file pone.0217270.s005.tif]

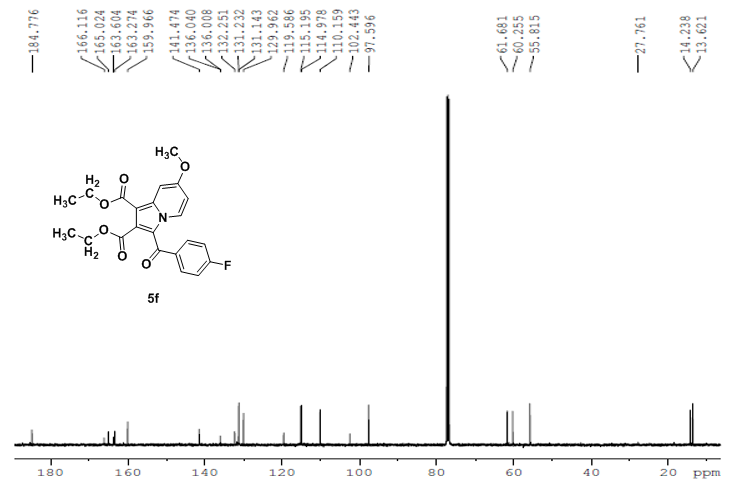

Supplement: S6 Fig — (TIF) [file pone.0217270.s006.tif]

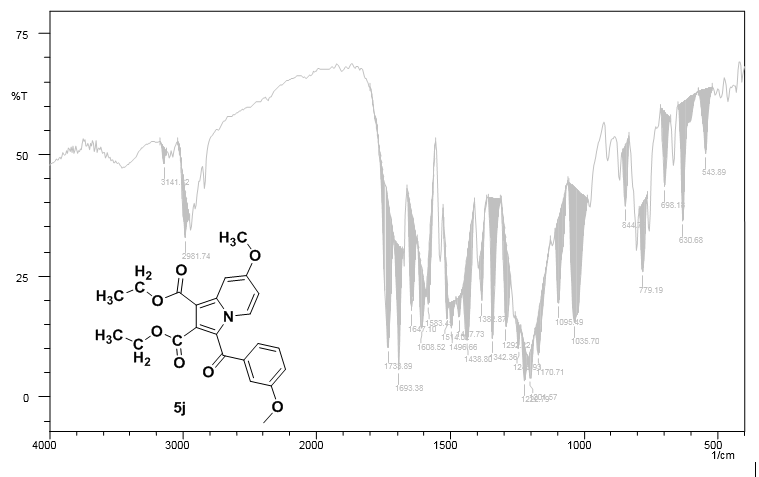

Supplement: S7 Fig — (TIF) [file pone.0217270.s007.tif]

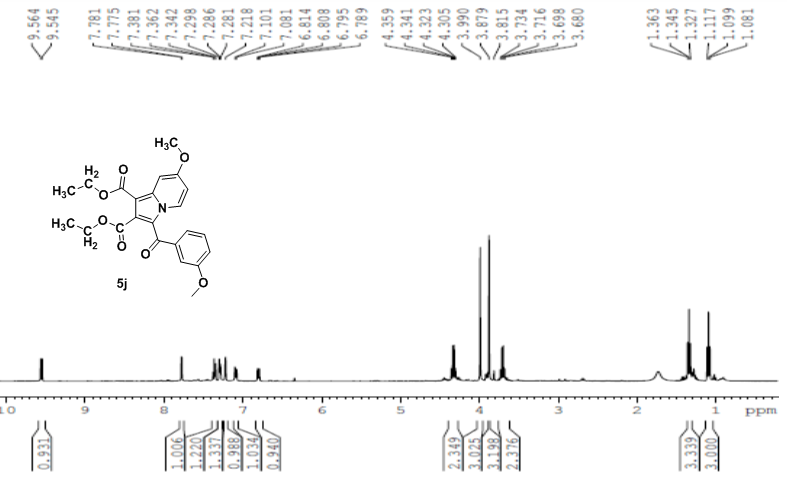

Supplement: S8 Fig — (TIF) [file pone.0217270.s008.tif]

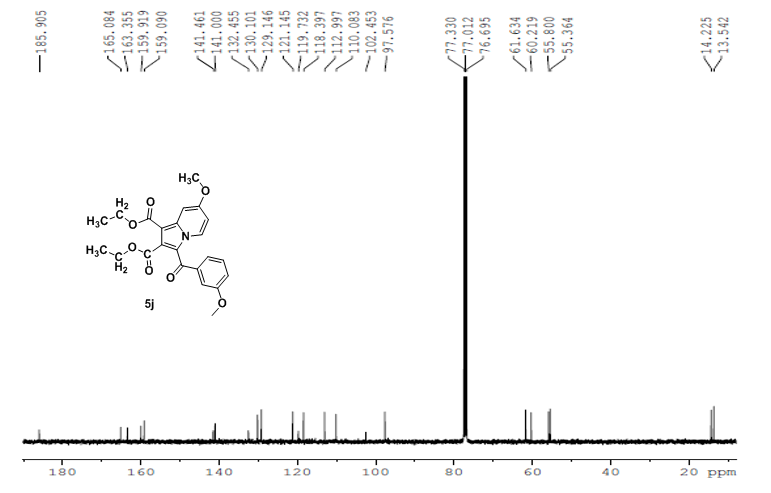

Supplement: S9 Fig — (TIF) [file pone.0217270.s009.tif]

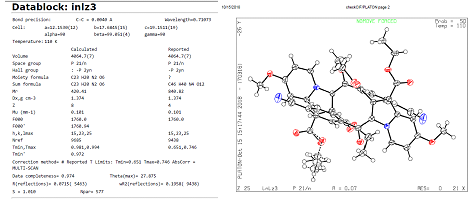

Supplement: S10 Fig — (TIF) [file pone.0217270.s010.tif]

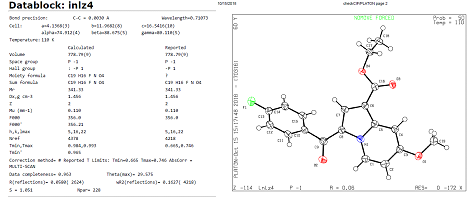

Supplement: S11 Fig — (TIF) [file pone.0217270.s011.tif]
